# The case study of prosthetic foot alignment on amputee gait kinematics using IMU-based motion capture system

**DOI:** 10.1080/23335432.2026.2663427

**Published:** 2026-05-01

**Authors:** Darya Korostovskaya, Tatyana Shashkina, Sofiya Eksharova, Vladimir Serdyukov

**Affiliations:** Biomechanics and Medical Engineering Laboratory, Novosibirsk State University, Novosibirsk, Russia

**Keywords:** Biomechanics, gait analysis, inertial measurement unit, transtibial amputation

## Abstract

The determination of the optimal prosthesis alignment for maximum patient comfort is an important rehabilitation goal, especially for lower limb amputees, where the assessment of biomechanics is complex. Despite this, quantitative gait analysis is rarely used in clinical practice, and the influence of prosthesis alignment on gait kinematics remains unclear. This study explores the use of an IMU-based motion capture system to assess the impact of prosthesis alignment on gait biomechanics. A transtibial amputee’s gait was analyzed with various prosthetic foot alignments in the sagittal and frontal planes. Key gait parameters were measured, including joint flexion/extension angles, body tilt and center of mass (COM) oscillation. Deviations in joint angles from reference values reached 30°, and gait speed varied by up to 0.5 m/s depending on alignment. Notably, prosthesis alignment had minimal impact on the healthy leg’s kinematics but significantly influenced gait asymmetry, also reflected in COM movement. The study confirms that prosthesis alignment can markedly affect amputee gait biomechanics. The proposed method enables clinicians to evaluate alignment suitability and make data-driven adjustments.

## Introduction

Today, the number of amputees in the world is steadily increasing due to traffic accidents, military injuries, musculoskeletal diseases, diabetes and other diseases, which may result in the loss of lower limbs or parts of them. According to estimates, the total number has reached nearly 58 million (McDonald et al. 2021), most of whom require rehabilitation aids. Along with proper prosthesis selection, precise adjustment is crucial for functionality and comfort, often requiring individualized monitoring. This issue is especially important for lower limb prostheses, where adjustment must consider anatomy, activity level, and gait biomechanics (Chow et al. 2006; Zhang et al. 2020). Improper prosthesis alignment may cause pain, musculoskeletal issues, and gait inefficiencies, while correct alignment improves mobility, quality of life, and energy efficiency (Schmalz et al. 2002; Fridman et al. 2003; Luza et al. 2020). Manufacturer guidelines often do not suffice for accurate prosthesis alignment, especially in complex cases like asymmetric residual limbs. In such situations, the prosthetist’s judgment is essential when adjusting alignments in different planes. Clinics are therefore seeking tools to objectively monitor the impact of prosthesis alignments on gait biomechanics.

Over recent decades, motion capture (Mocap) technologies such as optical systems and inertial sensors have advanced significantly. Optical systems provide high accuracy (Aggarwal and Cai 1999; Moeslund et al. 2006; Maletsky et al. 2007) through marker tracking and are widely used in gait biomechanics studies (Tang et al. 2015; Huang et al. 2024). However, optical Mocap systems are expensive, require complex calibration, and are prone to errors from marker occlusion. They are also limited to fixed environments, prompting the development of markerless AI-based systems (Takeda et al. 2021; Milone et al. 2024), which are still under refinement and not widely adopted in prosthetics.

In contrast, inertional measurement units (IMU)-based motion capture systems – using accelerometers, gyroscopes, and magnetometers – are gaining popularity due to mobility, lower cost, and ease of use. They are widely applied in virtual reality, sports and industry (Ahmad et al. 2013; Menolotto et al. 2020), and can reliably record high-frequency motion data, even outdoors. The study (Samala et al. 2024) shows that IMU systems effectively track sagittal plane motion in transtibial amputees, although they require regular calibration due to drift and magnetic interference. Numerous authors (Bastas et al. 2018; Rattanakoch et al. 2023) have demonstrated the viability of IMU systems for monitoring lower limb amputees gait and understanding prosthesis function. Although primarily used in research, these systems show strong potential for applied use in assessing prosthetic alignment in clinical alignments.

An analysis of the literature and regulatory and technical documentations has shown that there are currently no widely accepted international standards specifically dedicated to the assessment of rehabilitation of patients after lower limb amputation that comprehensively and explicitly include a section on biomechanical analysis of patient movements. Many international guidelines and protocols focus on general clinical outcomes, functional tests (e.g. the ”stand and walk” test, 6-min walk test) and quality of life assessment, while detailed quantitative gait biomechanics generally remain the subject of scientific research rather than a regulated standardized procedure. Therefore, in this context, the selected Russian GOST R 53871–2021 is a unique document that formalizes and mandates the use of objective biomechanical methods, such as stabilography and goniometry, to assess the effectiveness of prosthetics. This allows the rehabilitation process and the assessment of its results to be transferred from the realm of subjective conclusions to the realm of measurable and reproducible science.

The aim of this study was to evaluate the feasibility of using an IMU-based motion capture system to analyze how prosthesis alignment affects the gait of a transtibial amputee. Quantitative assessment of gait kinematics is required to substantiate its symmetry. Asymmetry in gait increases the likelihood of problems with the musculoskeletal system of amputees (Lloyd et al. 2010; Pröbsting et al. 2016). Quantitative assessment of gait kinematics allows for a more accurate assessment of gait symmetry than qualitative analysis of gait ‘by eye’, which is recommended in GOST. For a complete analysis of the gait kinematics, it is worth paying attention not only to the movement of the legs, but also to the position of the subject spine and center of mass (COM), since the walking directly affects the posture of the person. The tilt of the body can demonstrate how much the subject is trying to compensate for his unstable position on the incorrectly configured prosthesis.

## Methods

### Patient and prosthesis

Experiments to determine the effect of prosthetic alignment were conducted on one amputee. The patient was a 40-year-old man, height 180 cm and weight 88 kg, with a transtibial amputation (TTA) of the left leg. It is important to note that the amputee can be qualified as an experienced prosthesis user – he has about 6.5 years of experience in walking on a prosthesis, leads an active lifestyle, including participation in cyberathletic competitions. It can be stated that with the initial (basic) prosthesis alignment he does not have any pronounced movement problems. The subject was also interviewed and found to be most comfortable at a fast-walking pace.

The amputee uses a Freedom Agilix by Proteor prosthetic foot (size − 27, stiffness − 6). This prosthetic foot is classified as a K-3 activity level and is specially configured to absorb impacts vertically. It has been designed to manage loading impacts, reduce socket shear forces and improve comfort while walking on almost any terrain. According to the manufacturer, its dual split blade improves ground compliance and thus stability on any terrain.

### The prosthesis alignments

**Figure 1.**
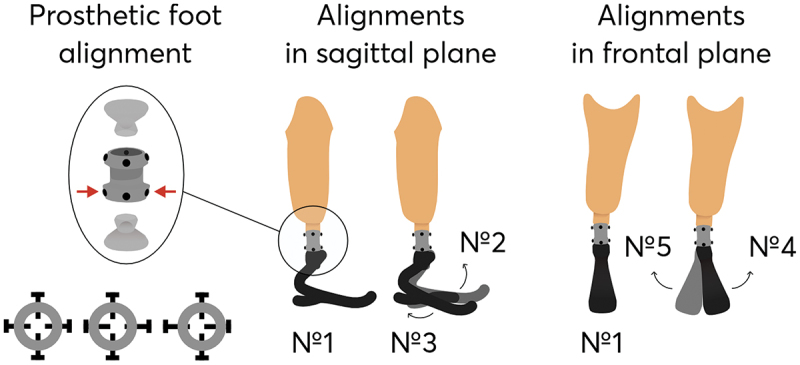
Scheme of the prosthetic foot alignments analyzed in the paper and their description.

In order to investigate the effect of prosthetic ankle alignment on the biomechanical parameters of amputee gait, the prosthesis was adjusted in the sagittal and frontal planes. For this purpose, the screws connecting the prosthesis to the pylon via pyramidal-type connector were screwed in and out (Figure 1). During the adjustment process, one of the axial screws was tightened and the other loosened. To change the alignment of the prosthesis, one or two complete turns of the corresponding screws were made clockwise or counterclockwise. Totally, five various alignments, including basics, were analyzed in the study, whose description is presented in Table 1.

**Table 1.** Description of the analyzed in the study prosthesis alignments.

Alignment №	Description
1	Basic alignment
2	2 turns forwards in the sagittal plane (dorsiflexed)
3	2 turns backwards in the sagittal plane (plantarflexed)
4	1 turn outwards in the frontal plane (valgus)
5	2 turns inwards in the frontal plane (varus)

### IMU system and sensors placement

**Figure 2.**
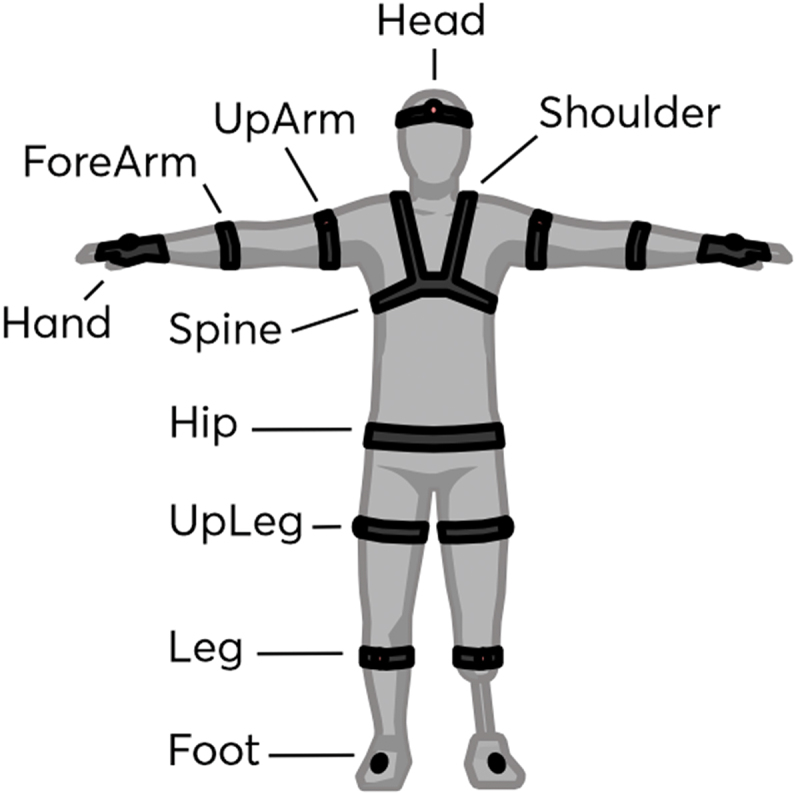
IMU sensors mounting diagram.

The IMU-based motion capture system Perception Neuron 3 (Noitom Ltd., Beijing, China) was used for data collection. Figure 2 shows the mounting scheme of the sensors. The 17 sensors were attached to the head, shoulder blades, back, pelvis, shoulders, forearms, hands, thighs, shins, and feet according to the manufacturer’s instructions. One sensor was placed on the head in the middle of the forehead, just above the eyebrows. Three sensors were placed on the back: one sensor was placed in the area of the Th3 vertebra, and two sensors were placed in the upper part of the shoulder blade area on the right and left sides. One sensor was placed on the sacrum in the pelvic area. Three sensors were placed on each arm (6 in total): 1 sensor is attached to the biceps of the shoulder, 1 sensor is attached to the broad part of the forearm along the line of the back of the palm, and 1 sensor is attached to the middle of the back of the palm. There are also 3 sensors on the left and right legs (6 in total): 1 sensor is located on the upper part of the outer surface of the thigh, 1 sensor is located on the front of the widest part of the lower leg, and 1 sensor is located on the back of the foot in the middle of the second and third metatarsal bones. This procedure is necessary to maximize the accuracy of data collection since the IMU system has a design property of being confounded due to the influence of environmental magnetic fields and due to sensor drift on clothing or skin.

The measurement uncertainties of the used IMU system are described in detail in Supplementary material 1.

### Data collection

Due to these peculiarities of the IMU system, the use of a treadmill was impossible, so during the recording the subject walked around the room at his comfortable pace. During each recording, the subject walked in a straight line for an average of 10 steps from the beginning to the end of the room, then turned around and walked back. The time of each data recording did not exceed 35 seconds, as this time is considered optimal for obtaining the most accurate data from the used IMU system (Choo et al. 2022). The data were recorded at 60 FPS. Data were also confirmed by video analysis (not video-based motion capture).

Each data set contains the relative joint angles of the corresponding body part in three planes and the coordinates of the COM. The sensor located on the sacrum tracks the movement of the center of mass in space. The other sensors calculate their position relative to it. The joint flexion angle and center of mass position data were imported directly from Axis Studio (the software for working with PN3), bypassing the conversion of accelerometer, gyroscope, and magnetometer data. The center of mass in Axis Studio is defined as the geometric center of mass, but this is sufficient for tracking its extreme positions and oscillation amplitudes.

For each prosthesis alignment, two data sets were recorded (a total of 10 for 5 alignments), containing 4 straight walks of 8–10 steps on both legs (4–5 steps on one leg). During data processing, each step from each straight walk of the patient was selected, and then these steps were averaged. *The step extraction algorithm and data processing scheme are shown in Supplementary material 2 Fig S1*. Thus, to average the data on the flexion/extension angles of the hip, knee, and foot of one leg, as well as the spine, arrays were collected, each containing 32 to 40 steps (gait cycles). The average flexion/extension angles of the spine in relation to the vertical axis were processed in the same way as the leg joint data.

The center of mass displacement data is known for the general human model in Axis Studio. The division of the center of mass position when stepping on the right and left legs is not considered to avoid errors in data processing. The set of minimum and maximum center of mass positions is a set of local minima and maxima from all data arrays for each alignment. Each set of minimums and maximums for a single alignment contains between 89 and 128 points, which is sufficient to track the spread of the position and amplitude of the COM oscillation.

### Study design

The study considered and analyzed basic biomechanical parameters (average walking speed and pace, stride length, and stride time), the hip, knee, ankle and back flexion/extension relative angles and the movement of the COM along the vertical Y-axis.

There are a number of common rehabilitation indicators that can be analyzed for the initial gait assessment of amputees. These indicators primarily reflect a person’s ability to walk independently and comfortably. It is often assumed that when these indicators meet normal parameters, the degree of rehabilitation is sufficient, and the patient can walk without additional support or assistance. In this study, general biomechanical indicators were compared with reference values for people with TTA, which were established in other studies (Skvortsov 1996; Schmid-Zalaudek et al. 2022), based on a sample of 362 people.

Gait kinematic analysis will help us understand how much prosthetic alignment affects joint flexion ranges, which, in turn, can increase the risk of secondary complications if the prosthesis is incorrectly aligned. In this case, the prosthetist’s recommendations and the patient’s perceived comfort are also taken into account.

The analysis of the variation of the extreme values of the COM position allows to study the gait symmetry. The minimum and maximum values of COM position are responsible for different phases of gait in both feet simultaneously. In the double support phase, the center of mass is at its lowest position. In the mid-swing phase, when the person’s weight rests entirely on one leg, the center of mass is at its highest position. In this position, one leg is supported, and the patient must balance on the other supporting leg. This is important because the amputee needs to be able to balance well on both the healthy leg and the prosthesis. The smaller the difference between the minimum and maximum COM values, the more symmetrical the gait.

An analysis of the patient’s back tilt during walking allows us to assess the potential for back pain due to improper prosthetic alignment. In this study, the spinal flexion angles of alignments № 2–5 were compared with those of alignment №1_basic, as the patient’s pre-amputation values were unknown and baseline alignment №1_basic was chosen as the reference.

### GOST data description

GOST describes clinical and biomechanical methods for assessing rehabilitation. The clinical assessment method is based on evaluating a person’s level of activity when using a prosthesis according to static and dynamic indicators. It mainly assesses a person’s ability to maintain certain positions and perform some basic necessary actions, such as walking, stability, ability to climb stairs, sit down, and so on. The clinical method also describes safety requirements and methods for assessing clinical indicators.

The biomechanical method describes the basic and spatiotemporal biomechanical parameters, as well as the kinematic and dynamic indicators of gait. The basic parameters include average walking speed and pace, stride length, and stride time. Spatiotemporal parameters include the time of the support and transfer phases, the double support phase, and the walking rhythm coefficient. In addition to the list of parameters, safety requirements, a description of the assessment methodology, and examples (not standards) of graphs of the conditional norm for hip, knee, and foot flexion angles in patients with transtibial amputation and transfemoral amputation are provided in comparison with healthy people. GOST does not specify the types of foot prostheses on which the studies were conducted and for which the sample graphs were constructed. GOST also does not specify the method of collecting joint flexion angle data, which makes it of little use for research. Therefore, from a methodological point of view, it would be more correct to compare the alignments of prostheses with each other rather than with GOST. The №1_basic prosthesis alignment will be taken as the reference gait, since it was selected by a prosthetist and is the most comfortable for the amputee.

## Results

### General biomechanical performance

The data obtained using the motion capture system on the general gait parameters of the amputee for the studied prosthesis alignments are shown in Table 2. The table demonstrates that with the exception of alignment №3_plantarflexed the gait parameters do not differ much from each other and in general correspond or are close to the specified norms. In turn, in the case of prosthesis alignment №3_plantarflexed, there is a significant decrease in walking speed, on average by 40% compared to the other alignments.

**Table 2.** Comparison of the obtained gait parameters with the reference data.

Alignment №	Walking speed (m/s)	Cadence (steps/min)	Step length (m)	Step Time (s)
1 (basic)	1.29	114.82	0.67	1.06
2 (dorsiflexed)	1.17	109.25	0.65	1.155
3 (plantarflexed)	0.765	104.7	0.63	1.14
4 (valgus)	1.26	112.05	0.63	1.035
5 (varus)	1.26	108.99	0.66	1.095
Data of (Schmid-Zalaudek et al. 2022)	–	103.78 ± 9.48	0.67 ± 0.06	1.16 ± 0.17

A comparison of the overall gait parameters with alignment №1_basic is provided. Alignment №3_plantarflexed differs most from the baseline alignment. Alignment №2_dorsiflexed also differs significantly from alignment №1_basic in gait parameters. Alignments №4_valgus and №5_varus differ least from the baseline alignment in most characteristics.

### Lower limbs joints flexion/extension angles analysis

**Figure 3.**
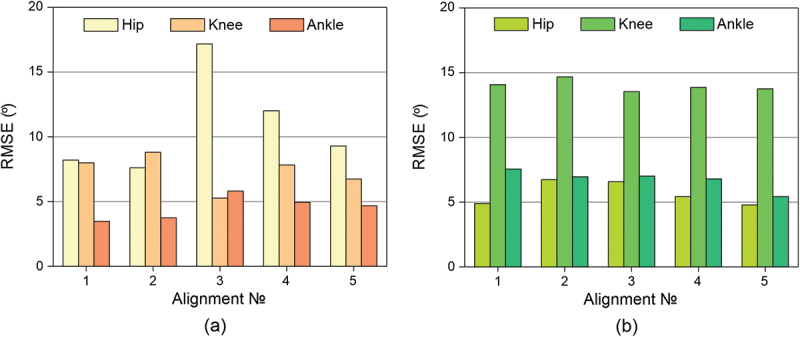
The RMSE deviations from the *GOST data* of the flexion/extension angles of the: (a) amputated (b) healthy leg.

**Figure 4.**
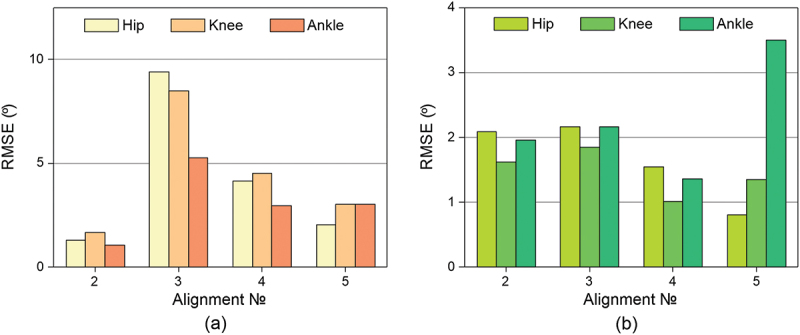
The RMSE deviations from the *basic alignment data* of the flexion/extension angles of the: (a) amputated (b) healthy leg.

Histograms of root mean square deviations (RMSE) were constructed for the obtained data for all alignments from GOST (Figure 3(a) for the amputated leg and 3b for the healthy leg) and alignments №2–5 from the №1_basic alignment (Figure 4(a) for the amputated leg and 4b for the healthy leg), recognized as the reference for this amputee.

#### RMSE for an amputated leg

Compared to the GOST: alignment №3_plantarflexed has the highest RMSE of the hip flexion angle, reaching 17 degrees, which is 1.5 times greater than that of other alignments, and the lowest RMSE of the knee flexion angle, reaching 5 degrees. The RMSE of the hip and knee flexion angles of other alignments reaches no more than 13 degrees.

Compared to basic alignments: the RMSE for alignment №4_valgus reaches 4.1, 4.5, and 2.9 degrees for the hip, knee, and ankle, respectively, while for alignment №5_varus, the deviation of the hip flexion angle reaches 2 degrees, and the knee and ankle flexion angles reach 3 degrees.

#### RMSE for a healthy leg

Compared to the GOST: RMSE for hip flexion angles is in the range from 5 to 7 degrees, for knee – from 13 to 15 degrees, for foot – from 5 to 7 degrees. All deviations are similar and differ little from each other.

Compared to basic alignments: RMSE for all joints is mainly in the range from 1 to 2.2 degrees, respectively. The exception is ankle flexion angles in alignment №5_varus, where RMSE reaches 3.5 degrees.

Averaged gait cycles were plotted for the hip, knee, and ankle for the amputated and healthy limb (see Supplementary material 3, Figures S2–S5).

### Analysis of average truncus angle and COM position

**Figure 5.**
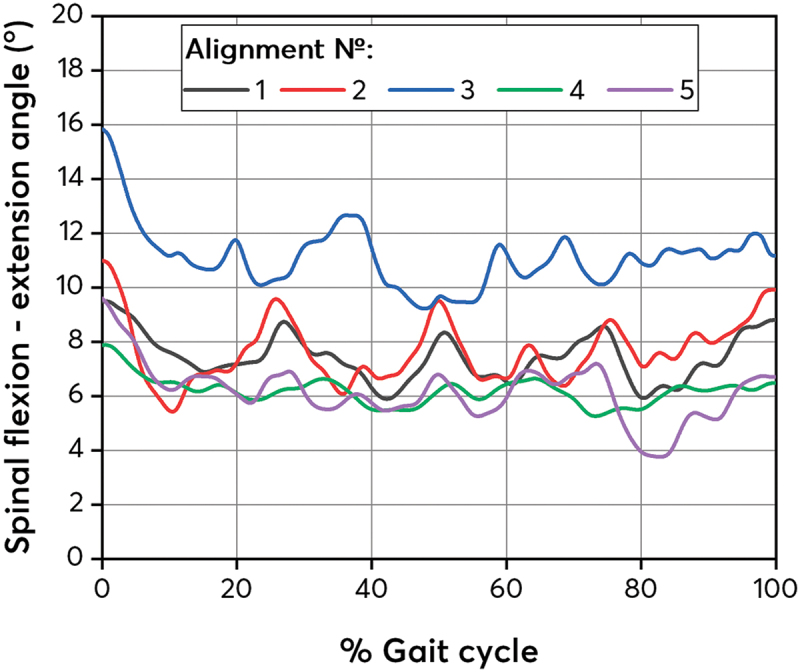
Average spinal flexion/extension angles for the studied prosthesis alignments.

Figure 5 shows the average flexion/extension angles of the spine in relation to the vertical axis. As mentioned previously in the Methods sectionin this case, the comparison of spinal flexion/extension angles is made relative to alignment №1_basic.

It can be seen that the amplitude of the subject body tilt changes as the prosthesis alignment changes. The largest amplitude is observed at alignment №2_dorsiflexed, with the amputee significantly tilts back and forth. The smallest amplitude of body tilt is observed for alignments №4_valgus and №5_varus, which indicates a more stable gait and less need to maintain balance by tilting.

It can also be seen that the angles of body tilt for alignment №3_plantarflexed are larger than for other alignments.

**Figure 6.**
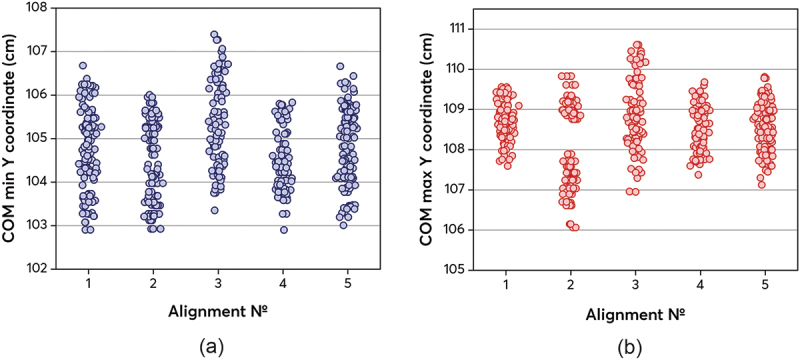
Center of mass extremum vertical coordinates: (а) minimums; (b) maximums.

Along with the body tilt, the COM movement relative to the vertical axis was considered (Figure 6). Figure 6 shows a strong gait asymmetry for alignment №2_dorsiflexed. In particular, in Figure 6(b) a clear partition of the COM maximums into 2 groups is observed. There is also a slight partition into 2 groups of minimums in Figure 6(a). This suggests that the patient seems to ‘bounce’ during gait. Because of this, alignment №2_dorsiflexed has a large amplitude of body tilt, which was demonstrated in Figure 5. Alignment №3_plantarflexed does not have a clear partition of data into 2 groups, as for alignment №2_dorsiflexed, but its data variation is also large.

In Figure 6(b), the minimum asymmetry is observed for alignments №1_basic, №4_valgus and №5_varus. However, the smallest point spread in Figure 6(a) is observed for alignment №4_valgus. Alignments №1_basic and №5_varus have a larger spread of minimum center of mass positions relative to alignment №4_valgus.

### Summarizing the results

For ease of summary, the patient gait biomechanics parameters obtained in the study for different prosthetic foot alignments can be presented in the form of a table (Table 3). However, it should be noted that there are no quantitative indicators or criteria for comparing these indicators in the literature today. In this case, the comparative result is presented relative to alignment №1_basic. The authors propose the following simple but illustrative metric:

1. « – » - a clear non-compliance with the criterion is detected;
2. « + » - the alignment falls within or close to the norms of the criterion;
3. « ++ » - the alignment has the best fit with the criterion among others.

**Table 3.** Summary of the study results.

Alignment №	Compliance with general biomechanical indicators (Table 2)	Analysis of flexion angles of joints (Figure 2)	Analysis of body tilt and COM position (Figures 3and 4 and undefined)
2 (dorsiflexed)	+	++	–
3 (plantarflexed)	–	–	–
4 (valgus)	++	–	++
5 (varus)	++	+	+

## Discussion

In this study, an IMU motion capture system was used to investigate the effect of different foot prosthesis positions on the gait kinematics of a person with a transtibial amputation. It should be noted that this system is not the gold standard for studying gait kinematics, but other studies have shown that its accuracy is sufficient for conducting such research. The study used five different prosthesis alignments, which were made in the sagittal or frontal planes.

The study used the GOST standard to compare the patient’s experimental data. Unfortunately, GOST does not specify the method of data collection and processing or the number of patients. It only provides a conditional norm for the angles of flexion of the hip, knee, and foot. Comparing experimental data with GOST data is not entirely correct from a methodological point of view, but GOST is the only standardized document in the world that is currently available in the public domain. Based on this, the present study compared the kinematics of the patient’s gait with different prosthesis alignments not only with GOST data, but also with the data of the patient’s №1_basic alignment, which was selected by the prosthetist and satisfied by the patient.

In the table with general biomechanical indicators (Table 2), the indicators for all alignments, except for alignment №3_plantarflexed, are within the normal range. This means that the patient experiences difficulties when walking. With severe plantar flexion, the patient noted that he was unable to fully land on his heel. To do so, he would have to lean back significantly, which increased the risk of losing balance and falling. He also noted that he could compensate for severe plantar flexion by bending forward in his back to push through the prosthesis and shift the weight from the toe to the heel. This behavior is also reflected in the spinal flexion/extension angle graphs, where alignment №3_plantarflexed achieves the highest values throughout the entire stride. In video recordings, plantar flexion causes the toe of the prosthesis to drop, resulting in a significant decrease in the angle between the foot and the floor at the beginning of the stance phase. This causes the prosthetic foot to roll more abruptly, requiring the patient to compensate with gait speed to maintain balance and avoid falling. In this case, general biomechanical indicators are insufficient to determine the effect of foot prosthesis alignment, so a more in-depth analysis of gait kinematics, back tilt, and center of mass displacement is required.

Comparing the flexion/extension angles of hip, knee and foot of all prosthesis alignments with GOST, it can be seen that the basic alignment has the lowest RMSE for almost every joint of both legs. It can also be seen that changing the alignment of the prosthesis has virtually no effect on the kinematics of the healthy leg, but there is a difference between the alignments on the amputated leg. Since №2_plantarflexed alignment has the greatest deviations from both GOST and the №1_basic alignment, the patient’s discomfort when walking with this prosthesis alignment is confirmed by the kinematics data.

A strong forward lean of the back while walking may indicate excessive work of the compensatory mechanisms to maintain balance. This position causes overload of the back muscles, which can lead to pain or deformation of the spine. As mentioned earlier, the greatest amplitude of spinal flexion is observed in №2_dorsiflexed alignment. №3_plantarflexed alignment also has the greatest asymmetry of gait, judging by the spread of the center of mass. This can cause constant back pain (Pröbsting et al. 2016) due to asymmetry and a large tilt of the spine. №2_dorsiflexed alignment also has a large amplitude of spinal flexion and a large spread of the center of mass positions, which can also negatively affect the patient’s musculoskeletal health.

It can be seen that the angle of spinal inclination and the spread of the center of mass positions increase when the prosthesis alignment is changed in the sagittal plane. Perhaps, when studying a larger number of people, a pattern will be found that reflects the dependence of the back tilt and the spread of the center of mass on the number of bolt rotations in the sagittal plane in the foot prosthesis head (№2_dorsiflexed/№3_plantarflexed alignment).

Finally, it should be noted that with the help of the IMU motion capture system, it is indeed possible to quantitatively track the difference between prosthesis alignments. However, for a complete analysis, it is necessary to study the dynamic characteristics of the gait of a patient with an amputated limb (e.g. energy expenditure) in order to obtain all the information about the characteristics of the gait on the prosthesis under study or its alignment. This requires data on the electrical activity of muscles obtained using electromyography (EMG) sensors. In addition, musculoskeletal modeling tools (such as OpenSim, Scone, etc.) should be used to study dynamic data that is difficult or impossible to obtain from experience.

It is also worth noting that the GOST standard definitely needs to be revised. It is necessary to include a specific methodology for data collection and processing. Further research plans include collecting a dataset from patients with transtibial amputation (approximately 30 individuals), averaging the data, and deriving conditional gait norms for use by prosthetists and rehabilitation specialists as a guide when assessing the course of rehabilitation or fitting and aligning a prosthesis.

## Supplementary Material

Supplemental Material
