# Supplementary material for: The case study of prosthetic foot alignment on amputee gait kinematics using IMU-based motion capture system
Source: Int Biomech. 2026 May 1;13(1):1–11. doi: 10.1080/23335432.2026.2663427 (PMC13137747; doi:10.1080/23335432.2026.2663427)
Supplement: Supplemental Material [file TBBE_A_2663427_SM8694.docx]

**Supplementary material**

1. ***Measurement uncertainties***

The IMU-based system manufacturer states that the data recording error depends on the position in which the data is captured. Thus, in the static position the error reaches 0.5 degrees when rotating and 1.5 degrees when tilting, in the dynamic position – 0.5 and 2 degrees, respectively. The manufacturer also states that data is captured at 600 Hz and transmitted at 60 Hz. The time delay is less than 20 ms, so we can consider the time error of data transmission to be insignificant. The manufacturer does not give an estimate of the error in recording COM movement data. However, based on experience in using the system, we can estimate this error to be ~3 cm.

There are also papers (Wu et al., 2022; He et al., 2024), in which the error of the IMU-based system data was calculated relative to the VICON marker optical motion capture system. The results showed, that the RMSE did not exceed 5° during walking and 8° during running. At the same time, the ankle RMSE can reach 22°, which is much higher than for other joints. Such errors are more consistent with reality, as they were manifested during the recording of experimental data.

Wu, Y., Tao, K., Chen, Q., Tian, Y., Sun, L., 2022. A comprehensive analysis of the validity and reliability of the perception neuron studio for upper-body motion capture. Sensors 22(18), 6954.

He, Y., Chen, Y., Tang, L., Chen, J., Tang, J., Yang, X., Su, S., Zhao, C., Xiao, N., 2024. Accuracy validation of a wearable IMU-based gait analysis in healthy female. BMC Sports Science, Medicine and Rehabilitation 16(1), 2.

1. ***Data collection and processing***

Gait cycles were extracted from each straight walk by finding local minima/maxima in the knee flexion/extension data array. After that, filtering of all cycles was performed by specifying the ranges of extrema of all cycles (Fig. S1). In this way, pass fragments corresponding to the amputee's reversal were cut off during processing. Then, the data were averaged – the coordinate average between all found cycles was determined.

The data was interpolated using the interp1d function from scikit python. In this case, linear data interpolation was performed when changing the size of arrays with gait cycles from 1 to 100%.


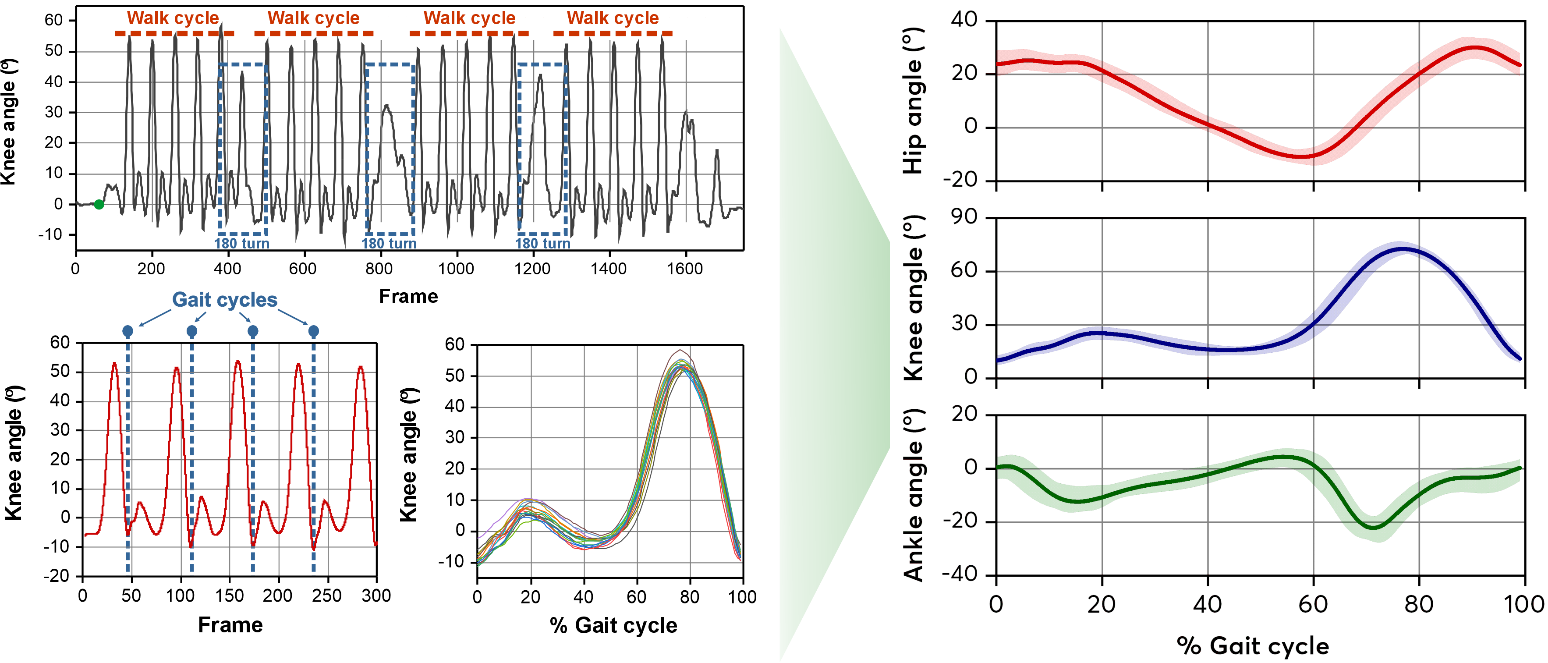


Fig. S1. The scheme of the experimental data on the joint flexion/extension angles processing process.

1. ***Lower limbs joints flexion/extension angles analysis***

Fig. S2 and S3 present averaged gait cycles for the hip, knee, and ankle for the amputated and healthy limb correspondently. For comparison, the corresponding data for patients with TTA according to GOST are also shown in the graphs. It is worth noting here that the description of the data from GOST does not specify the characteristics of the studied prosthesis (passive or active, degree of activity) that were used by the patients. However, the authors of the present paper assume that the data were obtained for a prosthesis of low activity level, which will be explained in detail when the ankle flexion angles of the amputated limb are discussed. In addition, the GOST does not detail the data on the walking speed of the analyzed amputees, which may lead to a slight difference in the timing of gait phases with the data of the present work.

First of all, it can be seen from the obtained data that the general appearance of the averaged plots for the amputated and healthy limb generally agrees with generally accepted walking dependencies, e.g. presented in Fukuchi et al., 2018. Thus, characteristic changes in the amplitude of flexion angles corresponding to different phases of gait are observed for all alignments. First, the general patterns of the experimental data for the amputated leg (Fig. S2) were analyzed.

It can be seen that the hip flexion/extension angle graphs for all prosthesis alignments show a pronounced peak at the end of the gait cycle. This is a consequence of the amputee personal walking peculiarities. As shown by the video recording of the patient walking, during the stance phase, before placing the ankle on the ground, the subject regularly raises the leg slightly, which is the reason for the extremum under discussion. In turn, for the knee flexion/extension angle data, there is a divergence of the extremes in time, which is likely a consequence of the difference between the subject velocities and the GOST data. It can also be noted that the first maximum extrema are not as clearly distinguished as in the GOST data. This is also due to the peculiarity of the amputee walking: in all cases he weakly bends the amputated leg during stance. An interesting dependence is observed for the ankle flexion/extension data for the amputee limb. It can be seen that the experimental data obtained differ significantly from the reference data. In particular, they are characterized by more pronounced extrema with large amplitudes of angles. These differences may be caused by the fact that the subject uses a prosthesis of high activity level made of carbon fiber, which is characterized by bending in the sagittal plane when stance on it. In turn, as noted earlier, it appears that the GOST data were obtained for a lower-activity prosthesis which material and construction are less flexible. For this reason, the high activity prosthesis used in this study mimics the behavior of the healthy ankle to a greater extent and its kinematics in the stance phase are similar to those of the healthy ankle. However, in the swing phase, the behavior of the prosthesis, even in the high activity phase, differs from the healthy ankle because the prosthesis does not flex during this phase, unlike the human ankle.

Comparison of the dependencies of flexion/extension angles of the amputated limb obtained for different prosthesis alignments shows that the position of the prosthesis significantly affects the gait kinematics. It can be seen that alignment №3 differs most strongly from the GOST data and the other alignments. The maximum deviation of hip flexion angles from GOST is achieved for this alignment, which means that the hip elevation during walking is too high. It is logical to assume that the higher a person has to lift the hip, the more energy will be spent on movement and the faster he will start to feel fatigue while walking. Therefore, such a large deviation is not good for the energy efficiency of walking. There is also the greatest compliance of the extremes of the knee flexion angles, which presumably arises from the low walking speed that the patient is uncomfortable with. Based on this, it can be confirmed that alignment №3 is not appropriate for the patient. Alignments №1 and №2 show the best result in terms of compliance with the reference data. In particular, they show the best compliance with the GOST hip and knee flexion angle. Alignments №4 and №5 show intermediate results relative to the others, from which it cannot be concluded that they are much better or worse than the other alignments.

At the next stage, the experimental data for the healthy limb were analyzed (Fig. S3). It can be seen, that there is a pronounced peak at the end of the gait cycle for all hip data, which is also caused by the peculiarity of the amputee gait described previously. For the knee and foot flexion data, there is a constant shift of extrema to the right in time relative to the reference dependence. This is due to the fact that the subject gait is characterized by asymmetry, and the swing phase of the healthy leg is smaller compared to the amputated one. In general, the averaged data for various alignments differ little from each other and have the same behavioral pattern. Thus, the kinematics of the healthy leg are not affected in any way by the different prosthesis alignments. This result is expected since the amputee is a fairly experienced user of a prosthetic foot.

**
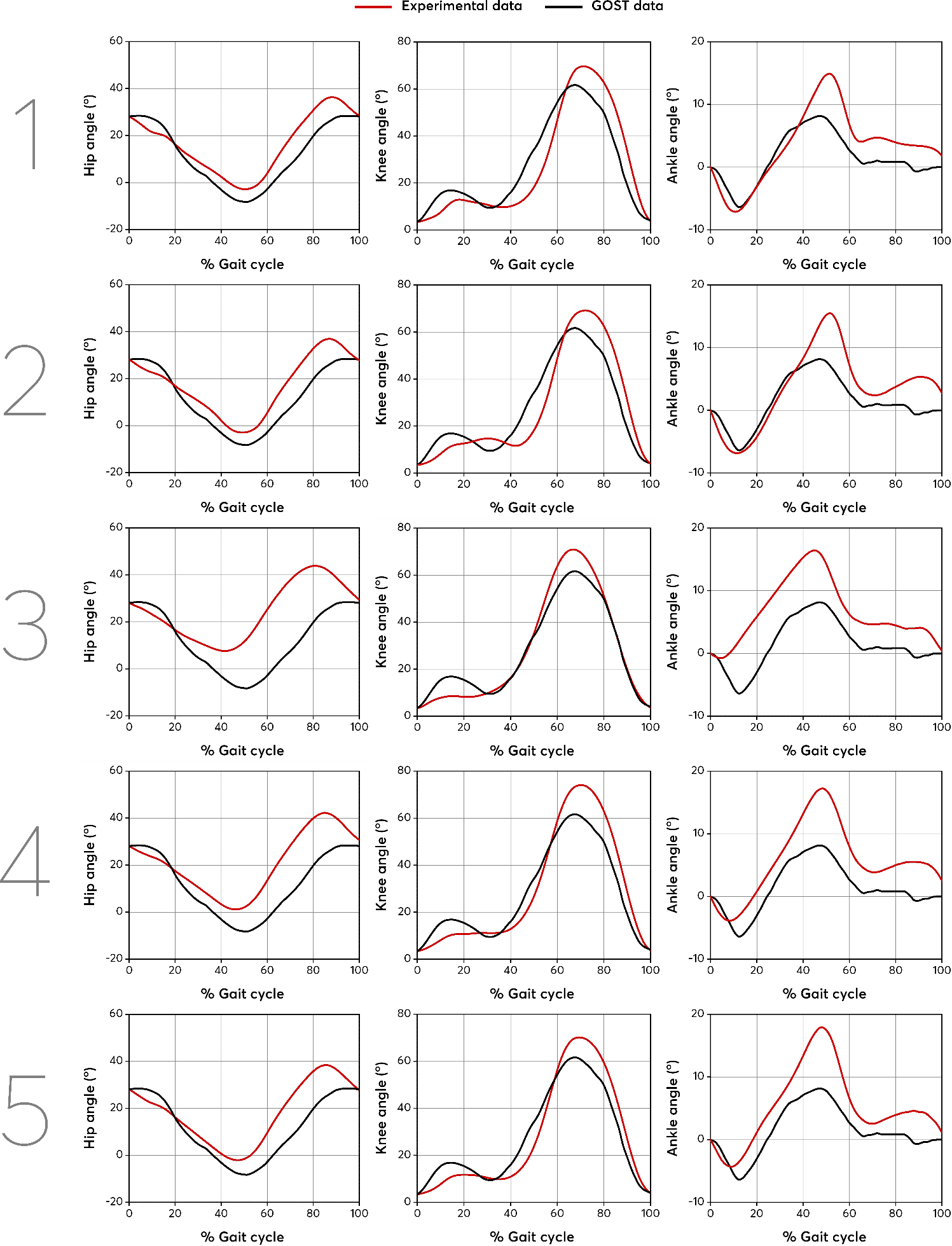
**

Fig. S2. Flexion/extension angles for the amputated leg joints at various prosthesis alignments.


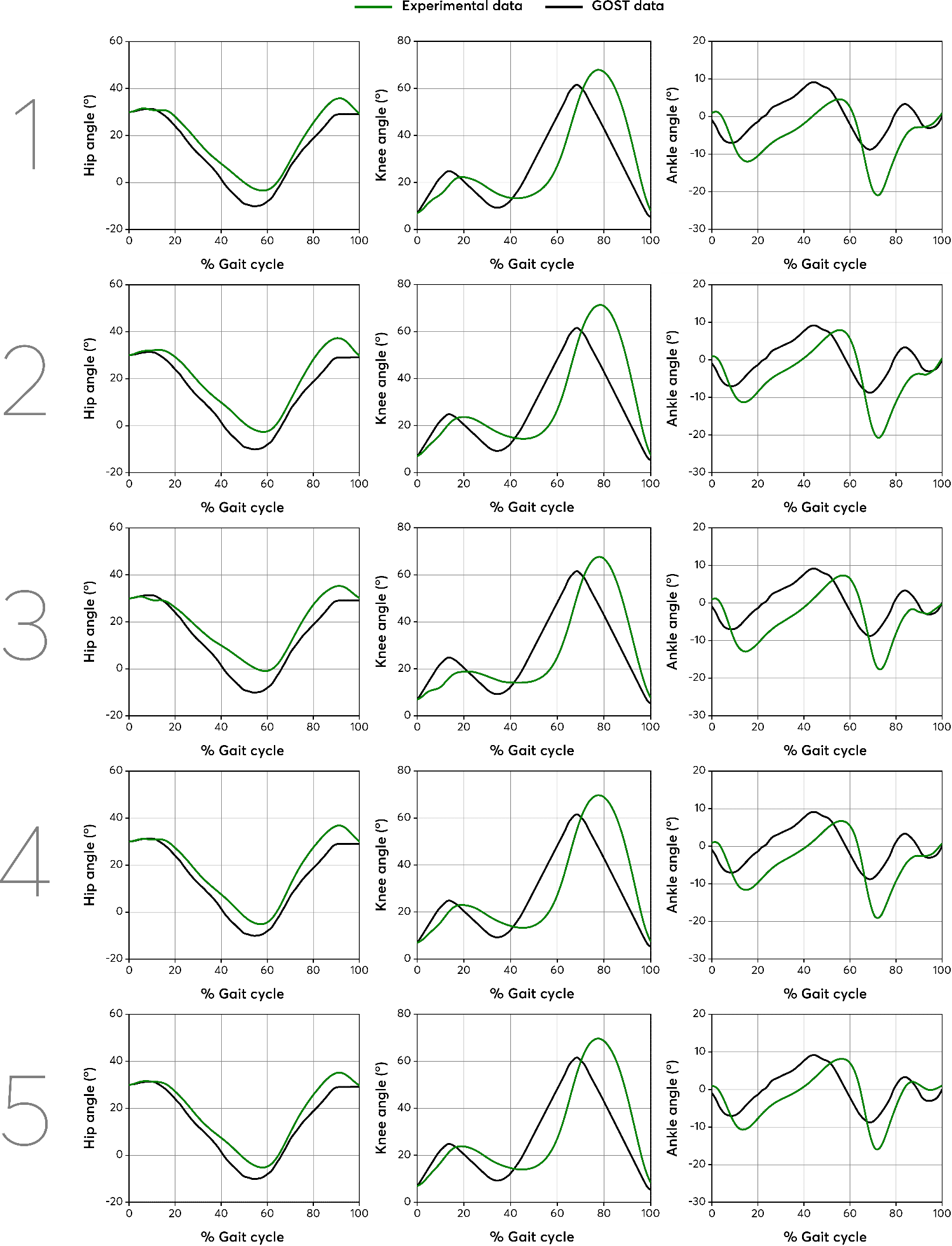


Fig. S3. Flexion/extension angles for the healthy leg joints at various prosthesis alignments.


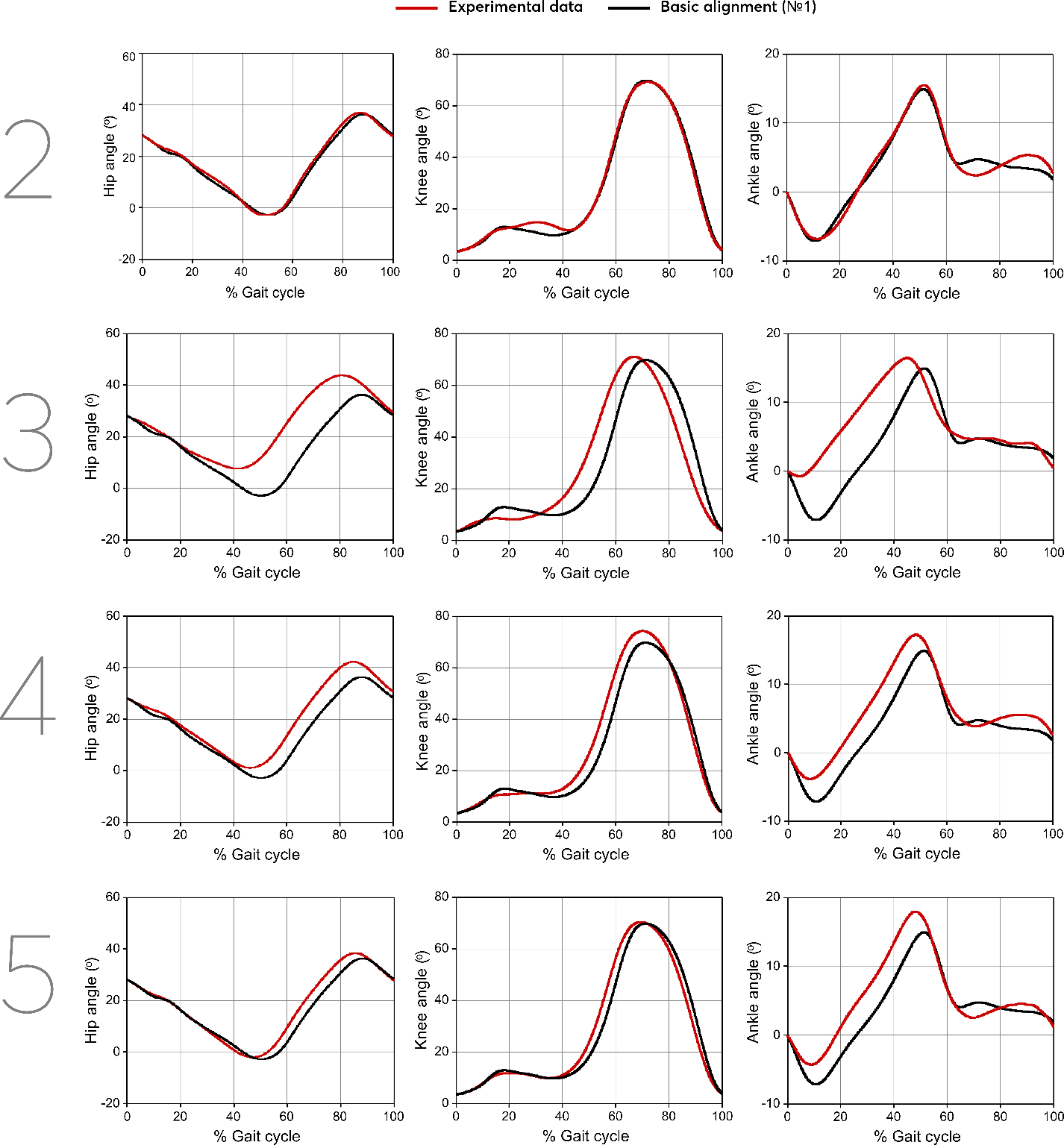


Fig. S4. Flexion/extension angles for the amputated leg joints at various prosthesis alignments (with basic alignment).


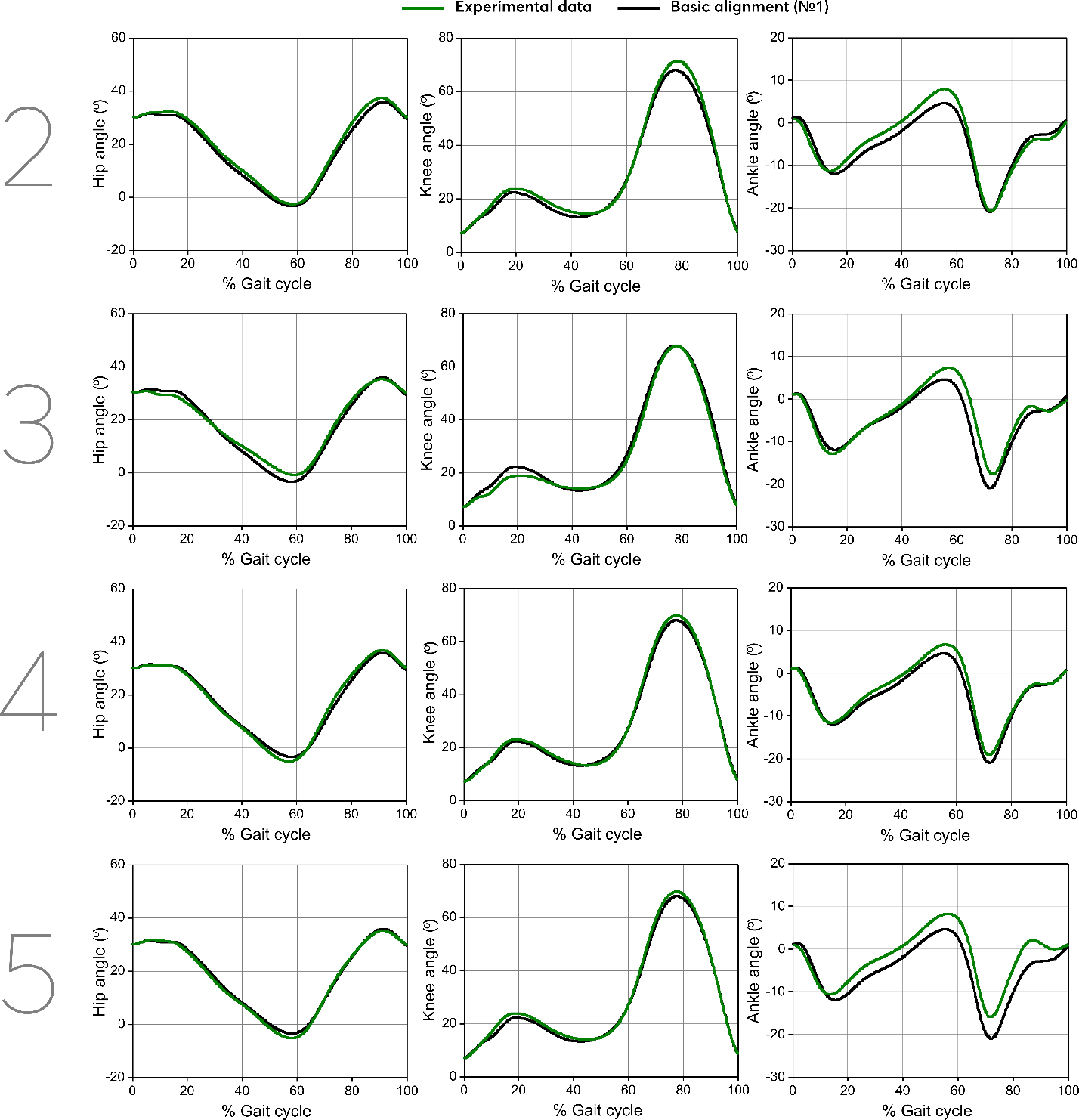


Fig. S5. Flexion/extension angles for the healthy leg joints at various prosthesis alignments (with basic alignment).
